# Case Report of Minimal Change Disease in the Adult Population

**DOI:** 10.7759/cureus.62306

**Published:** 2024-06-13

**Authors:** Austin B Wynn, Rajesh Metuku, Raul Santos

**Affiliations:** 1 Osteopathic Medicine, Alabama College of Osteopathic Medicine, Dothan, USA; 2 Nephrology, Archbold Memorial Hospital, Thomasville, USA; 3 Medical Education, Archbold Memorial Hospital, Thomasville, USA

**Keywords:** steroid-dependent minimal change disease, kidney biopsy, acute renal injury, adult onset, minimal change nephrotic syndrome (mcns)

## Abstract

Minimal change disease (MCD), typically linked with pediatric nephrotic syndrome, presents challenges in early identification and diagnosis in adult populations. This case report emphasizes the importance of tailored diagnostic and treatment approaches for adults with MCD. Our patient presented with fatigue, shortness of breath, and confusion, along with other symptoms leading to a renal biopsy which confirmed MCD. This highlights the diagnostic significance of kidney biopsy in adults. While steroids remain the standard treatment, challenges such as resistance and side effects lead to the consideration of alternatives like tacrolimus. There are nuanced differences between adult and pediatric MCD presentations, for which our study calls for increased awareness among physicians. Steroids are considered a first-line treatment for MCD, but prolonged use of steroids has significantly increased risk and alternative therapies should be considered. This study presents an example of MCD in adult populations, urging ongoing research for enhanced understanding and tailored management strategies. It emphasizes the pivotal role of physician awareness, alternative treatments, and continued investigation to improve outcomes for adults with MCD.

## Introduction

Minimal change disease tends to be associated with children presenting with nephrotic syndrome. However, minimal change disease is something physicians need to consider in adult populations as they usually present with atypical symptoms and at a lower frequency. With most research and treatment modalities based on children, this case report works to give more insight into adult-onset minimal change disease in terms of how they present, the treatment doses, and options to consider. Minimal change disease is a nephrotic condition that presents with proteinuria, particularly the loss of albumin, edema, hyperlipidemia, and hypercoagulability [1]. While minimal change-disease in children can be confirmed by symptoms and a urinalysis, adults can present atypically, so a kidney biopsy might be required to confirm the diagnosis, as seen with our patient. While minimal change disease can be idiopathic or by secondary causes, the pathophysiology is thought to be based on T-lymphocytes specifically regulatory T-cells [2]. Regulatory T-cells have the main purpose of maintaining homeostasis along with being able to differentiate between self and foreign antigens [2]. It was thought that either regulatory T-cell dysfunction and/or impaired autoregulatory function of the podocyte could inactive CD80, leading to proteinuria and eventually to minimal change disease [2]. Diagnosis can be confirmed by kidney biopsy under an electron microscope, which will show the effacement of podocyte foot processes on the glomerular basement membrane [3]. The loss of podocytes weakens the negative charge barrier, allowing albumin to more easily leak into the urine and cause nephrotic syndrome [4]. The mainstay treatment for MCD for both adults and children is steroids which resolve the symptoms and the condition [3]. If a patient is unable to take steroids or the disease is steroid-resistant, alternative drugs such as cyclophosphamide, cyclosporine, tacrolimus, or even rituximab can be used [4, 5]. Relapse rates are higher in adults than children likely due to increased steroid resistance. Side effects from prolonged steroid use include obesity, metabolic syndrome, and cosmetic changes such as “moon facies” [6, 7]. These side effects can reduce adherence to treatment and increase mortality in the affected patients. With patients and physicians concerned with the long-term effects of steroids, tacrolimus is a good alternative treatment for de novo minimal change patients with similar efficacy of steroids [7]. Tacrolimus is classified as a calcineurin inhibitor that suppresses interleukin-2 (IL-2) transcription, inhibiting T-cell activation responsible for minimal change in disease [7].

## Case presentation

A 60-year-old Caucasian male with no significant past medical history presented with a one-week onset of increasing shortness of breath and fatigue. The patient’s family member endorsed increasing confusion over the past week. Previous chest x-ray and pulmonary function tests the week prior showed no abnormalities. Serum lab work and urinalysis, seen in Table 1 below, show low complement levels and an acute kidney injury seen with the glomerular filtration rate (GFR), blood urea nitrogen (BUN), and creatinine levels.

**Table 1:** Serum lab values and Urinalysis The table shows severe kidney dysfunction with loss of complement and increased light chains. Urinalysis further showed a massive loss of protein in the urine. GFR: glomerular filtration rate.

Serum labs	Patient's Value	Reference Value
eGFR	4.82	90-120 mL/min/1,73m^2^
Blood urea nitrogen	104	6-20 mg/dL
Creatinine	13.6	0.8-1.2 mg/dL
Complement 3	52	88-201 mg/dL
Complement 4	5	15-45 mg/dL
Lambda light chain	364.2	5.7-26.3 mg/L
Kappa light chain	548.8	3.3-19.4 mg/L
Kappa/Lambda light chain ratio	1.51	0.26-1.65
Urinalysis		
Microalbumin	>4400	30-300 ug/mL
Random protein	>600	10-20 mg/dL
Urine protein/creatinine ratio	2.7	<30 grams

**Figure 1:**
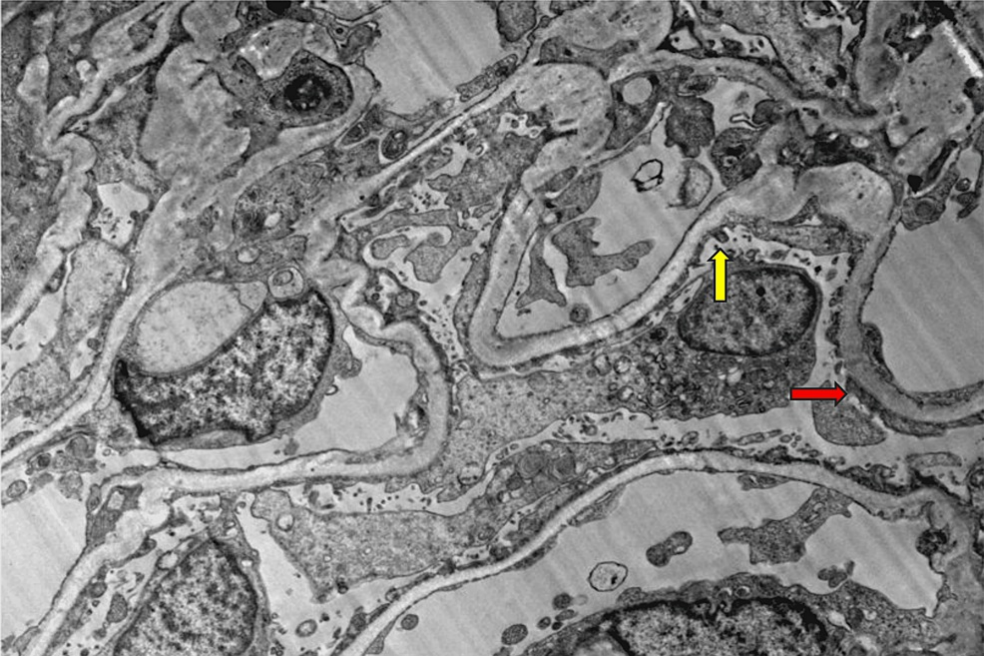
Electron microscopy of kidney cortex The red arrow indicates podocyte foot process effacement seen at the glomerular basement membrane. Yellow arrow indicating normal podocyte within the cortex.

Renal ultrasound was unremarkable. The patient was admitted for further management and treatment of oliguric acute renal failure of unknown etiology and concurrent metabolic acidosis with interventional radiology (IVR) Vas-Cath placement the following day. The patient underwent three consecutive treatments of dialysis with improvement of renal function. Vas-Cath was ultimately removed. A core renal biopsy was performed and ultrastructural evaluation of a glomerulus revealed uniform basement membranes with no increased thickness. Glomerular capillary loops were patent. No immune-type electron-dense deposits were present along the glomerular basement membranes. There was severe epithelial foot process effacement with segmental microvillous transformation seen in Figure 1.

**Figure 2:**
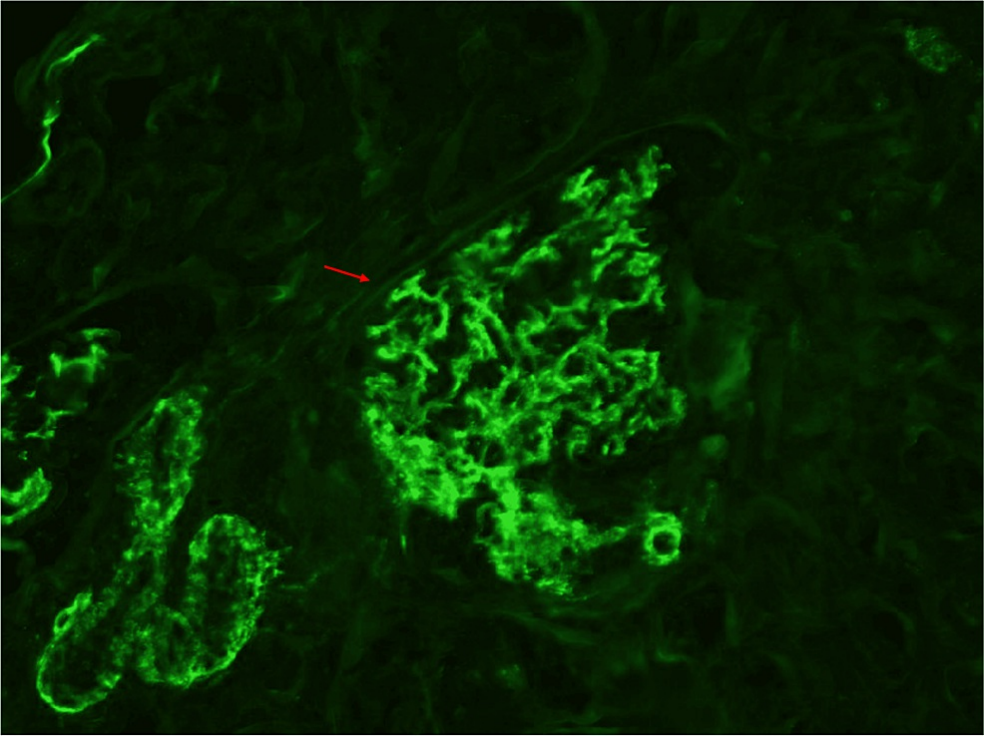
Negative immunofluorescence of kidney cortex Red arrow showing diffuse spread of immunofluorescence of a glomeruli. There is mesangial granular staining for IgA (1+), IgG (trace), C3 (3+), kappa (1+), and lambda (1+)

The mesangium had rare ill-defined electron densities. The tubular basement membranes were without deposits and there was immunofluorescence staining as seen in Figure 2.

After the renal biopsy, the patient did have perirenal bleeding post-biopsy and underwent coil embolization of the arcuate artery, which controlled the bleeding. Pulse dose steroids were given for three days and prednisone 60 mg was started and tapered over time. Anasarca, characterized as severe generalized fluid accumulation, did develop during the hospital stay, which was managed with diuretics. The patient then followed up with nephrology for continued management of minimal change disease.

## Discussion

This case report gives insight into how physicians should approach and treat minimal change disease in adults, a population that presents atypically and rarely at all. Documenting and reporting on such cases gives physicians a better idea of how to approach these cases. The presentation of minimal change disease varies between adults and children with adults likely to present with unique symptoms and variable outcomes. Our patient's initial presentation symptoms of fatigue, shortness of breath, and confusion were present for one week and it was the abnormal renal panel that shifted our focus to the kidney as the culprit. With an atypical presentation and a reduced GFR indicating acute renal failure, hemodialysis is used to clear toxins as seen in our case. With all other lab reports being inconclusive, it was the kidney biopsy that showed the hallmark presentation of minimal change disease. Steroids were administered as they have a long track record of reversing the effects of minimal change disease and preventing any long-term complications [1]. The use of steroids is standard treatment in minimal change disease as it is thought to cause remission in 80% of affected adults with many other studies confirming its effectiveness [4,6,8]. However, adult outcomes in minimal change disease are not as positive as adult populations have a higher risk for relapse and steroid resistance. The relapse rate is higher among adults with 56-76% relapsing at least once [4]. Often, the prognosis is worse with possible focal segmental glomerulosclerosis (FSGS) on repeat kidney biopsy with alternative treatments, like tacrolimus, considered more often [4]. Fortunately, our patient did well with the initial steroid therapy. The patient began to produce adequate urine output and renal indices began to improve back toward the patient's baseline status of a creatinine of 1.0 mg/dL. While minimal change disease is a rare disease in adults, there are previous recorded case reports that go into detail about the condition and the treatment options that were used. One such case includes a patient with shortness of breath, lower limb edema, and a 30-pound weight gain [9]. This patient also had elevated creatinine levels and urinalysis demonstrating proteinuria due to some unknown renal pathology, necessitating a biopsy. Renal biopsy showed the loss of the foot process proving it to be minimal change disease in which treatment was started. The initial treatment was intravenous (IV) steroids which did not work until the patient was diuresed well and that was when the steroid became effective and that dose was continued. Further acute kidney injury lead to the addition of tacrolimus [9]. Ultimately, various research studies and case reports prove that steroids are effective in treating minimal change for the most part with the possibility of switching to another immunosuppressive on a case-by-case situation. Overall, researching more about this condition and presenting more case reports to the public about this condition helps us better understand this condition and how to effectively treat it.

## Conclusions

The appearance of minimal change disease in the adult population is rare, accounting for 10% to 15% of primary nephrotic syndromes in adults, and documenting and reporting each case helps us better understand this condition in adults. With its atypical presentation compared to MCD in children, it is harder to diagnose in adults. A kidney biopsy may be a first-line option for diagnosing MCD in adults, as seen in our patient. While MCD is thought to involve T-cell dysfunction, more research is needed to confirm which part of the immune system is involved as there are multiple theories and possible etiology that can lead to MCD. This case report should highlight one example of atypical symptomatology of MCD in adult populations to aid in earlier detection of the disease. Further understanding of the pathophysiology of MCD could lead to easier recognition of the disease state, allowing for early intervention, before permanent damage to the kidneys can occur.
